# Synaptic Connections of Aromatase Circuits in the Medial Amygdala Are Sex Specific

**DOI:** 10.1523/ENEURO.0489-19.2020

**Published:** 2020-06-17

**Authors:** Addison Billing, Marcelo Henrique Correia, Diane A. Kelly, Geng-Lin Li, Joseph F. Bergan

**Affiliations:** Neuroscience and Behavior Program, Department of Psychological and Brain Sciences, University of Massachusetts, Amherst, Amherst, Massachusetts 01003

**Keywords:** accessory olfactory bulb, aromatase, chemical cues, circuitry, medial amygdala, sex difference

## Abstract

The brains of male and female mice are shaped by genetics and hormones during development. The enzyme aromatase helps establish sex differences in social behaviors and in the neural circuits that produce these behaviors. The medial amygdala of mice contains a large population of aromatase neurons and is a critical hub in the social behavior network. Moreover, the neural representation of social stimuli in the medial amygdala displays clear sex differences that track developmental changes in social behaviors. Here, we identify a potential anatomic basis for those sex differences. We found that sensory input from the accessory olfactory bulb (AOB) to aromatase neurons is derived nearly exclusively from the anterior AOB, which selectively responds to chemosensory cues from conspecific animals. Through the coordinated use of mouse transgenics and viral-based circuit-tracing strategies, we demonstrate a clear sex difference in the volume of synapses connecting the accessory olfactory bulb to aromatase-expressing neurons in the medial amygdala of male versus female mice. This difference in anatomy likely mediates, at least in part, sex differences in medial amygdala-mediated social behaviors.

## Significance Statement

The medial amygdala is a central hub of the social behavior network of the brain that integrates social information and produces behaviors like aggression, parenting, and reproduction. We determined that in mice, medial amygdala neurons expressing aromatase, an enzyme that converts testosterone to estradiol and plays an important role in establishing neuroanatomical sex differences, receive sensory information from a restricted population of pheromone-sensitive neurons in the vomeronasal pathway. These aromatase-expressing neurons had similar intrinsic electrophysiological properties in both sexes but received more sensory inputs in males than in females. We propose that the different anatomic configurations of social circuits described here contribute to known sex differences in medial amygdala function and, ultimately, to sex differences in critically important social behaviors.

## Introduction

**Figure 1.**
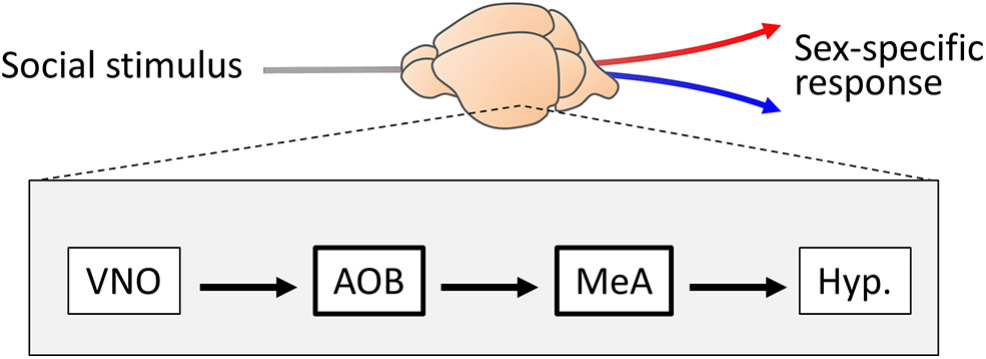
Circuit contributions to sex differences in social behavior. Top, Sex differences in behavior indicate that the same sensory stimulus produces different responses in males and females. Bottom, The vomeronasal system is a critical mediator of social behaviors that often display sex differences. Arrows (bottom) indicate direct anatomic connections between brain regions. Hyp, Hypothalamus.

Mice broadcast signals critical for social behavior using semiochemicals that are detected by the vomeronasal organ (VNO) and relayed via the accessory olfactory bulb to the social behavior network (Stowers et al., 2002; Mandiyan et al., 2005). The medial amygdala (MeA) serves as a hub for neural circuits within the social behavior network that identify social cues and guide innate social behaviors (Newman, 1999). These social behaviors are central for an individual’s survival and rely on conserved neural circuits (Tinbergen, 1951; Petrovich et al., 2001; Goodson, 2005). MeA-mediated social behaviors including parenting, aggression, mating, and defense are often sexually differentiated (Ferguson et al., 2001; Unger et al., 2015; Yao et al., 2017), implying that sex differences exist in the configuration and function of the underlying MeA circuits (Fig. 1; Lehman et al., 1980; Wu et al., 2009). But precisely which features differ in these circuits and how do they produce different patterns of behavior?

We investigated the configuration of aromatase-expressing (arom^+^) neurons in the MeA because of their critical role in establishing and maintaining sex differences in social behaviors. We hypothesized that the arom^+^ neurons of MeA would either receive sexually differentiated sensory input or that the intrinsic properties of arom^+^ neurons would be different in male and female mice. Here, we demonstrate that arom^+^ neurons of both sexes receive input near exclusively from the anterior division of the accessory olfactory bulb (AOB), with few inputs from the posterior AOB. We also observed no sex difference in the intrinsic electrophysiological function of arom^+^ neurons. However, the synaptic convergence from the AOB to individual arom^+^ neurons is substantially greater in males than in females. Together, these data suggest that the volume of synaptic connections made between the AOB and arom^+^ neurons in the MeA provides an anatomic basis for sexually differentiated function in the MeA.

## Materials and Methods

*Animals.* Forty-four male and female adult mice (10–16 weeks old) were group housed in single-sex cages in a temperature-controlled (22°C) and light-controlled (12 h light/dark) facility, with *ad libitum* access to food and water. All animal procedures were approved by the University of Massachusetts at Amherst Institutional Animal Care and Use Committee and were performed in compliance with all animal care regulations.

The Cyp19a1-Cre transgenic line was generated by BAC (bacterial artificial chromosome) recombination (Yao et al., 2017). A Lox-STOP-Lox-tdTomato reporter line with a LoxP-flanked STOP was used to express tdTomato in Cre^+^ cells (Ai9, The Jackson Laboratory; Madisen et al., 2010). Homozygous Cyp19a1-Cre mice were crossed with homozygous rosa26-lsl-tdTomato reporter mice to visualize aromatase-expressing neurons. The resulting double transgenic mice expressed bright tdTomato fluorescence consistent with past studies investigating aromatase expression in the brain. Faithful genetic access to aromatase neurons using this strategy was previously described and verified in the study by Yao et al. (2017).

**Figure 2.**
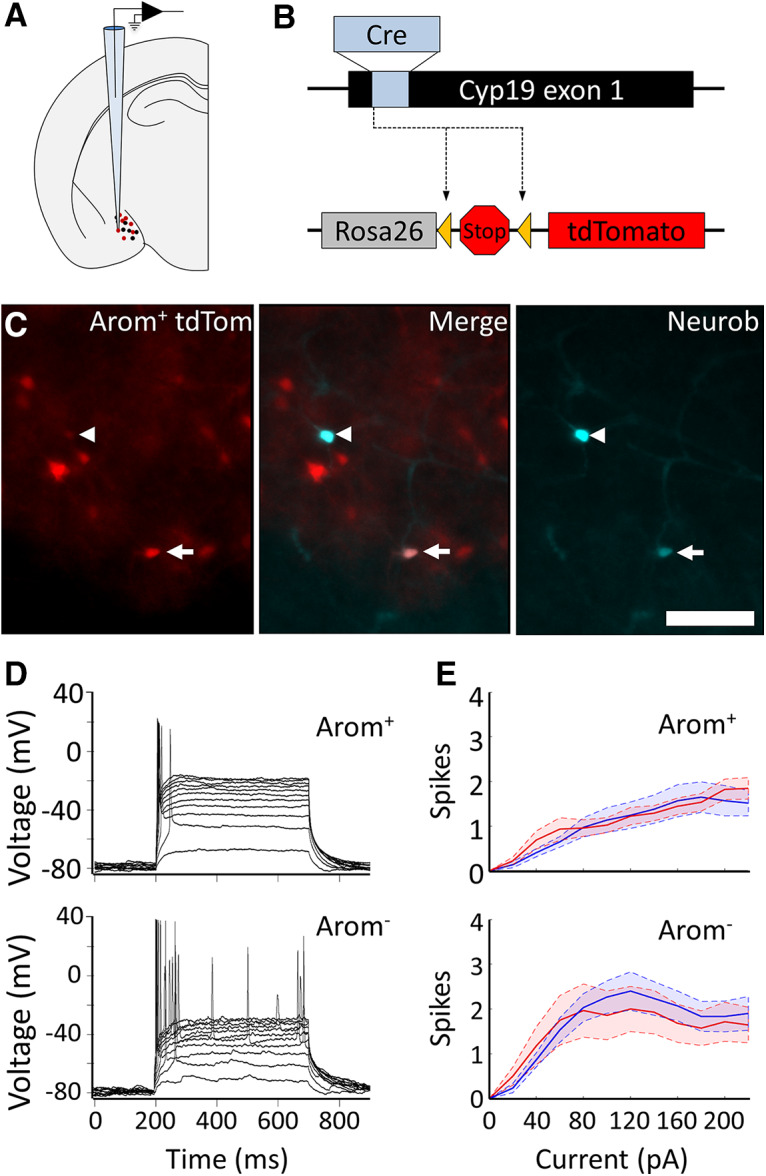
*Ex vivo* characterization of the intrinsic electrophysiological properties of arom^+^ and arom^−^ neurons in males and females. ***A***, Schematic of whole-cell patch recording strategy from the MeA of transgenic mice. ***B***, Arom^+^ neurons were distinguished from arom^−^ neurons based on tdTomato expression in a cross between a transgenic mouse line expressing Cre in arom^+^ neurons (Yao et al., 2017) and a tdTomato reporter line (Madisen et al., 2010). ***C***, Left, Aromatase-expressing neurons are shown in red. Right, Two recorded neurons filled with neurobiotin-streptavidin are shown in cyan. Middle, Merged channels showing one recorded neuron was arom^+^ (arrow) and the other was arom^−^ (arrowhead). Scale bar, 250 μm. ***D***, Firing pattern of a single arom^+^ neuron (top) and arom^−^ neuron (bottom) in response to current steps ranging from 20 to 220 pA. ***E***, Average number of spikes are shown for male (blue) and female (red) MeA neurons, with arom ^+^ neurons (top) and arom^−^ neurons (bottom). Shaded areas indicate SEM.

*Slice electrophysiology.* Whole-cell patch recordings were made from the posterior dorsal MeA in adult male and adult female (age, 10–16 weeks) mouse brain slices (Fig. 2*A*). Female mice in the diestrus phase of the estrus cycle were used. Animals were killed the hour before lights-off, and slice recordings were performed during the first 4 h of the dark phase of the 12 h light/dark cycle in the vivarium. The arom^+^ neurons were distinguished from neurons that did not express aromatase (arom^−^) based on the expression of tdTomato in arom^+^ neurons. Briefly, targeted expression of tdTomato was achieved in arom^+^ neurons by crossing an aromatase-cre transgenic mouse (Yao et al., 2017) with a tdTomato reporter line (Fig. 2*B*; Madisen et al., 2010). Fluorescent arom^+^ neurons were easily distinguished from arom^−^ neurons, and all recorded neurons were filled with neurobiotin for *post hoc* confirmation of cell type, morphology, and location (Fig. 2*C*).

Mice were deeply anesthetized with isoflurane and killed. The brain was rapidly removed and placed in ice-cold (bubbling 95% O_2_/5% CO_2_) solution containing the following: 89.1 mm sucrose, 13.88 mm glucose, 87.27 mm NaCl, 25 mm NaHCO_3_, 2.5 mm KCl, 7 mm MgCl_2_/6H_2_O, 0.37 mm CaCl_2_, and 1.25 mm NaH_2_PO_4_ in H_2_O. Coronal slices (300 μm) were cut using a vibratome (model VT1200S, Leica) and incubated at 35°C for 30 min in oxygenated artificial CSF (ACSF) containing the following: 127 mm NaCl, 25 mm glucose, 25 mm NaHCO_3_, 2.5 mm KCl, 1.325 mm MgCl_2_, 2.5 mm CaCl_2_, and 1.2 mm NaH_2_PO_4_ in H_2_O. Slices were then transferred to a recording chamber.

Slices were perfused with oxygenated ACSF and maintained at room temperature for the duration of the recording session. Electrodes were made with borosilicate glass drawn from a vertical pipette extractor (PC-10, Narishige) and were filled with an internal solution (20 mm KCl, 120 mm KGlu, 0.1 mm CaCl_2_, 5 mm EGTA, 5 mm HEPES, 3 mm MgATP, and 0.5 mm NaGTP, at pH 7.3 osmolarity of 290 mOsm, and impedence of 8–12 MΩ). Whole-cell patch-clamp recordings were made from arom^+^ neurons identified by tdTomato expression and arom^−^ neurons in the MeA using infrared differential interference contrast and an upright fluorescent microscope (BX51WIF, Olympus) with a 60× water immersion lens. Neurobiotin (0.1%) was added to the internal solution to fill the recorded neurons for subsequent visualization and morphologic analysis.

After achieving whole-cell configuration, voltage was clamped at −80 mV, and a “soft” current-clamp switch was performed in which the current used to hold the cell at −80 mV was maintained. Neurons were then depolarized by injecting current ranging from 20 to 220 pA in 20 pA steps each for a duration of 500 ms. Recorded voltage signals were filtered and then sampled at 100 kHz. The number of action potentials elicited by each current step was recorded for each neuron. The baseline spike rate was determined by injecting no current and recording for 1 min. The threshold for eliciting an action potential was determined by ramping current injection from 0 to 200 pA over a 1 s epoch; the threshold was determined by the timing of the first spike during this epoch. Measurements were calculated using Igor Pro 6.0 software (WaveMetrics) and MATLAB (MathWorks). Following each recording, brain slices were fixed in PFA for 2 h at room temperature, washed three times in PBST (0.1% Triton X-100 in PBS, pH 8) for 15 min, and incubated in fluorescently conjugated streptavidin (1:200; DyLight 649, Vector Laboratories) to visualize the recorded neurons. After 1 h, sections were washed three times in PBS, mounted on slides, and imaged using a BX51WIF upright fluorescent microscope (Olympus).

*Stereotaxic injections.* Iontophoretic injections of the anatomic tracer Fluoro-Gold (10% in double distilled H_2_O; fluorochrome) were made in the posterior dorsal MeA of male and female mice (bregma, −1.8 mm; lateral, 1.8 mm; depth, 4.5–5.5 mm; retaining current, −1 μA constant DC; injection current, +5 μA constant DC; 10 min). Anesthesia was maintained with 1–2% isoflurane in aseptic conditions throughout the surgery. Good retrograde labeling was observed after a survival period of 7 d. Animals were deeply anesthetized with isoflurane and exsanguinated with 50 ml of cold PBS followed by 25 ml of cold PFA (4% in PBS). The brain was extracted and postfixed in 4% PFA at 4°C overnight. Fluoro-Gold-labeled tissue samples were sectioned coronally (100 μm) and visualized with an ultraviolet excitation filter.

**Figure 3.**
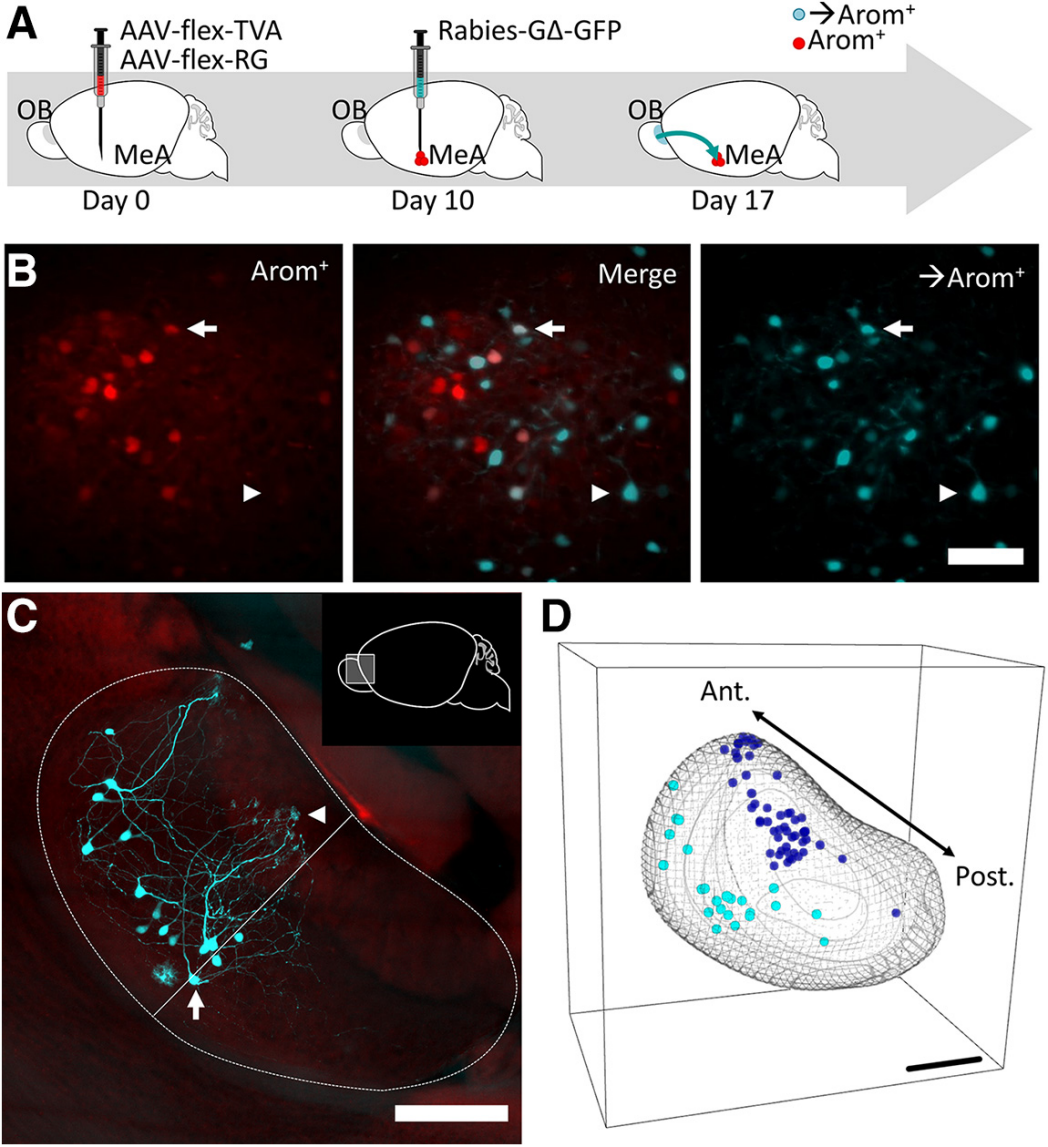
Strategy for circuit mapping from arom^+^ neurons in the MeA. ***A***, Conditional AAV vectors were used to express TVA, RG, and mCherry in arom^+^ neurons of the MeA (Materials and Methods). After 10 d, G-deleted rabies (GFP) was injected at the same stereotaxic coordinates to infect arom^+^ starter neurons, after which 7 d were allowed for rabies to be transported from arom^+^ neurons in the MeA to the AOB. ***B***, Horizontal image of MeA showing arom^+^ neurons (red) and arom^+^ →arom^+^) in cyan (arrowhead). Colabeled neurons were interpreted as starter neurons (arrow). ***C***, The AOB was rendered transparent and imaged intact. A maximum intensity projection of the entire AOB showing →arom^+^ neurons monosynaptically labeled from the ipsilateral MeA. Arrow, Labeled mitral cell; arrowhead, labeled dendritic arbor in glomerulus; linea alba, thin straight line. ***D***, 3D representation of all →arom^+^ neurons in the AOB in a single animal (cyan, cell bodies; blue, glomeruli). Note the strong anterior bias of both cell bodies and glomeruli. Scale bars, 250 μm.

We next used an approach based on a modified rabies virus to identify monosynaptic input specific to the arom^+^ neurons in the MeA (Wickersham et al., 2007; Watabe-Uchida et al., 2012; Menegas et al., 2015). Briefly, a nonendogenous cell-surface receptor (TVA) required for rabies infection was coexpressed with a rabies envelope glycoprotein (RG) required for replication in arom^+^ neurons of the MeA (Fig. 3*A*).

All adeno-associated viruses (AAVs) were produced by the UNC Vector Core Facility (Chapel Hill, NC). Rabies circuit tracing experiments involved two consecutive stereotaxic injections in both sexes. In the first injection, 500 nl of AAV-FLEX-TVA and AAV-FLEX-RG (both serotype 8; mixed 1:1; Watabe-Uchida et al., 2012) were injected into the posterior dorsal MeA of aromatase-cre mice (bregma, −1.8; lateral, 1.8; depth, 4.5–5.5). After 14 d, 300 nl of SADΔG-EGFP(EnvA) virus (Viral Vector Core, Salk Institute, La Jolla, CA) was injected into the MeA at the same stereotaxic coordinates (Wickersham et al., 2007). In two animals, 250 nl of AAV-FLEX-TVA (without AAV-FLEX-RG) was injected and, after 14 d, was followed by a 300 nl injection of SADΔG-EGFP(EnvA) virus in the aromatase-cre tdTomato reporter double transgenic line. Excluding AAV-FLEX-RG prevents the synaptic spread of the SADΔG-EGFP(EnvA) virus and allowed us to confirm colocalization of initial rabies infection to cre-expressing (tdTomato-positive) neurons. This is consistent with published reports showing the high genetic specificity of the rabies-based strategy (Wickersham et al., 2007; Watabe-Uchida et al., 2012; Menegas et al., 2015).

Ten days after the final injection, animals were deeply anesthetized with isoflurane and exsanguinated with 50 ml of cold PBS followed by 25 ml of cold PFA (4% in PBS). The brain was extracted and postfixed in 25 ml of hydrogel (recipe: 40 ml of 40% acrylamide, 10 ml of 2% bis-acrylamide, 1 g of VA-044 initiator, 40 ml of 10× PBS, 100 ml of 16% PFA, 210 ml of H_2_O; Chung et al., 2013) at 4°C for 48 h.

After 48 h of incubation in hydrogel, oxygen was flushed from the hydrogel by bubbling the liquid hydrogel solution with nitrogen. The tissue container was resealed and transferred to a 37°C water bath for 1 h. If hydrogel was not yet solidified, the sample was returned to the water bath until polymerization was complete. Excess hydrogel was removed from the brain manually, and the tissue sample was washed in PBST (0.1% Triton X-100 in PBS, pH 8) overnight at 37°C with gentle rocking. The brain was then transferred to clearing solution and passively incubated for 2 d before active clearing.

Active clearing was performed in a 5 gallon bucket with a custom magnetohydrodynamic (MHD) clearing device (Joseph F. Dwyer, unpublished observations). Briefly, the tissue sample was placed at the intersection of a strong magnetic field and a 0.3 A (∼30 V) electrical field. The resulting MHD force rapidly removed unbound lipids from the tissue sample and also circulated the clearing solution to keep the sample at a constant temperature. Brains were cleared until bright white and translucent, which typically took 48 h. Brains were then transferred to PBST (0.1% Triton X-100, pH 8) for 24 h with three changes of the PBST solution to help remove the remaining clearing solution. Brains were transferred from PBST to an OptiView imaging solution (Isogai et al., 2017) with a refractive index of 1.45 and incubated at 37°C for 2 d before imaging.

AOB and MeA images were acquired with the Zeiss Z.1 Lightsheet Microscope (Carl Zeiss). Rabies-labeled GFP^+^ neurons were excited with a 488 nm laser, and a 561 nm laser was used to produce an autofluorescence image for subsequent background subtraction and isolation of the GFP signal. Images were collected with a 5× objective (MeA) or 20× objective (AOB) with PCO-Edge scMOS cameras (PCO). The MeA was imaged horizontally from the ventral surface of the brain, and the AOB was imaged sagittally from its lateral edge. Images were saved at 1–5 μm resolution for cell counting, fiber mapping, and outlining of brain regions. ImageJ was used to format images into .tif stacks.

*Data analysis.* Statistical analyses and figure generation were done using validated MATLAB scripts (MathWorks). Slice electrophysiology comparisons between two groups were performed with an unpaired *t* test and a mixed-model ANOVA for nested comparisons of more than two groups. Statistical results were considered significant at *p *<* *0.05, and ANOVAs returning a significant *p* value were followed by pairwise comparisons with Bonferroni correction to investigate the significant differences. Cohen’s d was calculated by finding the difference of means between each group and dividing by the pooled SD. All means are reported with SE measurements. Regressions were performed in MATLAB using the *fitlm* and *predict* functions. All statistical tests are presented in Table 1 along with a more complete description of the statistical results.

**Table 1:** Summary of statistical analyses

	Data structure	Type of test	Sample size	Statistical data
a	Normal distribution	Unpaired two-sample *t* test	Arom^+^ neurons: *n* = 46 male, 47 female	*p* = 0.36, *t* = 0.91, df = 82
b	Normal distribution	Unpaired two-sample *t* test	Arom^+^ neurons: *n* = 46 male, 47 female	*p* = 0.27, *t* = 1.12, df = 76
c	Normal distribution	Unpaired two-sample *t* test	Arom^−^ neurons: *n* = 31 male, 34 female	*p* = 0.83, *t* = 0.22, df = 57;
d	Normal distribution	Unpaired two-sample *t* test	Arom^−^ neurons: *n* = 31 male, 34 female	*p* = 0.32, *t* = 1.01, df = 54
e	Normal distribution	Mixed-model ANOVA: (current step, sex of animal)	*N* = 14 male mice; *N* = 15 female mice	df = 10,1,10; *F*(current) = 3.32,*F*(sex) = 2.8, *F*(interaction) = 0.29;*p*(current) = 0.0004, *p*(sex) = 0.095,*p*(interaction) = 0.98
f	Normal distribution	Mixed model ANOVA: (current step, sex of animal)	*N* = 14 male mice; *N* = 15 female mice	df = 10,1,10; *F*(current) = 2.37,*F*(sex) = 0.43, *F*(interaction) = 0.34;*p*(current) = 0.01, *p*(sex) = 0.51,*p*(interaction) = 0.97
g	Normal distribution	Mixed model ANOVA: (current step, cell type: arom^+^ vs arom^−^)	*N* = 14 male mice; *N* = 15 female mice	df = 10,1,10; *F*(current) = 4.75,*F*(cell type) =9.78, *F*(interaction) = 0.91;*p*(current) < 0.00001, *p*(cell type) = 0.0018,*p*(interaction) = 0.52
h	Normal distribution	Unpaired *t* test	*N* = 14 mice (6 female; 8 male)	p < 0.00001, *t* = 17.6, df = 20
i	Normal distribution	Unpaired *t* test	*N* = 7 mice	*p* = 0.41, *t* = 0.88, df = 8
j	Normal distribution	Unpaired *t* test	*N* = 14 mice (6 female; 8 male)	*p* < 0.00001, *t* = 21.4, df = 16
k	Normal distribution	Unpaired two-sample *t* test	*N* = 8 male mice; *N* = 6 female mice	*p* = 0.93, *t* = 0.08, df = 12
l	Normal distribution	Unpaired two-sample *t* test	*N* = 8 male mice; *N* = 6 female mice	*p* = 0.04, *t* = 2.22, df = 12
m	Normal distribution	Unpaired two-sample *t* test	*N* = 8 male mice; *N* = 6 female mice	*p* = 0.24, *t* = 1.2, df = 9

## Results

Because aromatase expression is critical to establish sex differences in MeA function, we hypothesized that the intrinsic electrophysiological properties of arom^+^ MeA neurons may be different in male and female mice. To test this hypothesis, we measured the electrophysiological properties of arom^+^ neurons and arom^−^ neurons in brain slices from 14 male and 15 female mice (Fig. 2*D*; arom^+^ neurons: *n* = 46 male, 47 female; arom^−^ neurons: *n* = 65, 31 male, 34 female). In contrast to our hypothesis, we found no evidence for sex differences in the intrinsic electrophysiological properties of MeA neurons. No sex difference was observed for arom^+^ neurons for either input resistance (male, 740 ± 60 MΩ; female, 670 ± 40 MΩ; unpaired *t* test: *p* = 0.36, *t* = 0.91, df = 82; Table 1, a) or the threshold voltage for eliciting an action potential (male, −45.9 ± 1.4 mV; female, −47.9 ± 0.95 mV; unpaired *t* test: *p* = 0.27, *t* = 1.12, df = 76; Table 1, b). Similarly, no sex difference was observed for arom^−^ neurons for either input resistance (male, 680 ± 70 MΩ; female, 700 ± 70 MΩ; unpaired *t* test: *p* = 0.83, *t* = 0.22, df = 57; Table 1, c) or the threshold voltage for eliciting an action potential (male, −49.4 ± 1.5 mV; female, −53.4 ± 1.25 mV; unpaired *t* test: *p* = 0.32, *t* = 1.01, df = 54; Table 1, d). In addition, when we measured the number of action potentials elicited by current steps, we observed no significant effect of sex on the number of current-evoked spikes in arom^+^ neurons (Fig. 2*E*, top; mixed-model ANOVA: *F* = 2.8, df = 1, *p* = 0.10; Table 1, e; Cohen’s d for maximum number of spikes, 0.19), or the number of current-evoked spikes in arom^−^ neurons of male versus female mice at any current (Fig. 2*E*, bottom; mixed-model ANOVA: *F* = 0.43, df = 1, *p* = 0.52, Table 1, f; Cohen’s d for maximum number of spikes, 0.20). However, arom^−^ neurons produced more action potentials than arom^+^ neurons in response to the same current step (Fig. 2*D*,*E*; ANOVA: *F* = 9.78, df = 1, *p* = 0.002, Table 1, g). Moreover, the firing rate of arom^−^ neurons increased more steeply with increasing current steps, with a peak firing rate for injected current of 80 pA followed by a gradual reduction in response to larger current steps (Fig. 2*E*).

Next, we used a modified rabies viral tracing strategy to investigate the configuration of arom^+^ neural circuits (Wickersham et al., 2007). Briefly, initial infection was restricted to arom^+^ neurons based on cre-dependent expression of the TVA receptor, which is required for EnvA pseudotyped rabies virus to infect mammalian cells, in arom-cre mice. Trans-synaptic spread to neurons that contact arom^+^ neurons was made possible by also expressing the rabies envelope RG only in arom^+^ neurons (Fig. 3*A*). We identified both local neurons with direct projections to arom^+^ neurons (Fig. 3*B*, Movie 1), as well as long-range input from the AOB, which we will abbreviate as →arom^+^ neurons (Fig. 3*C*,*D*, Movie 2). No neurons were labeled in the main olfactory bulb. The →arom^+^ neurons were located in the mitral/tufted cell layer (Fig. 3*C*, arrow), and clear dendritic arborizations were visible in AOB glomeruli of the AOB (Fig. 3*C*, arrowhead).

Movie 1. Visualization of viral injection site in the MeA. Horizontal view of the MeA showing tdTomato in arom^+^ neurons (red, aromatase-cre X tdTomato reporter line) and GFP (green) in neurons infected with the modified SADΔG-EGFP(EnvA) rabies virus. Both cell bodies and neural processes are visible. Each new frame represents a movement of 1 μm into the brain starting near the ventral surface of the MeA. The field of view was 0.96 mm in both dimensions. 10.1523/ENEURO.0489-19.2020.video.1

Movie 2. Visualization of rabies-labeled neurons in the AOB. Sagittal view of the AOB showing GFP (green) in neurons infected with the modified SADΔG-EGFP(EnvA) rabies virus that project to arom^+^ neurons in the MeA. An autofluorescence image was also collected (excitation, 561 nm) for improved visualization (red). Cell bodies, neural processes, and dendritic arborization in the AOB glomeruli are visible. Each new frame represents a movement of 1 μm into the brain starting near the lateral surface of the AOB and moving medial. Anterior is to the left. The field of view was 1.3 mm in both dimensions. 10.1523/ENEURO.0489-19.2020.video.2

**Figure 4.**
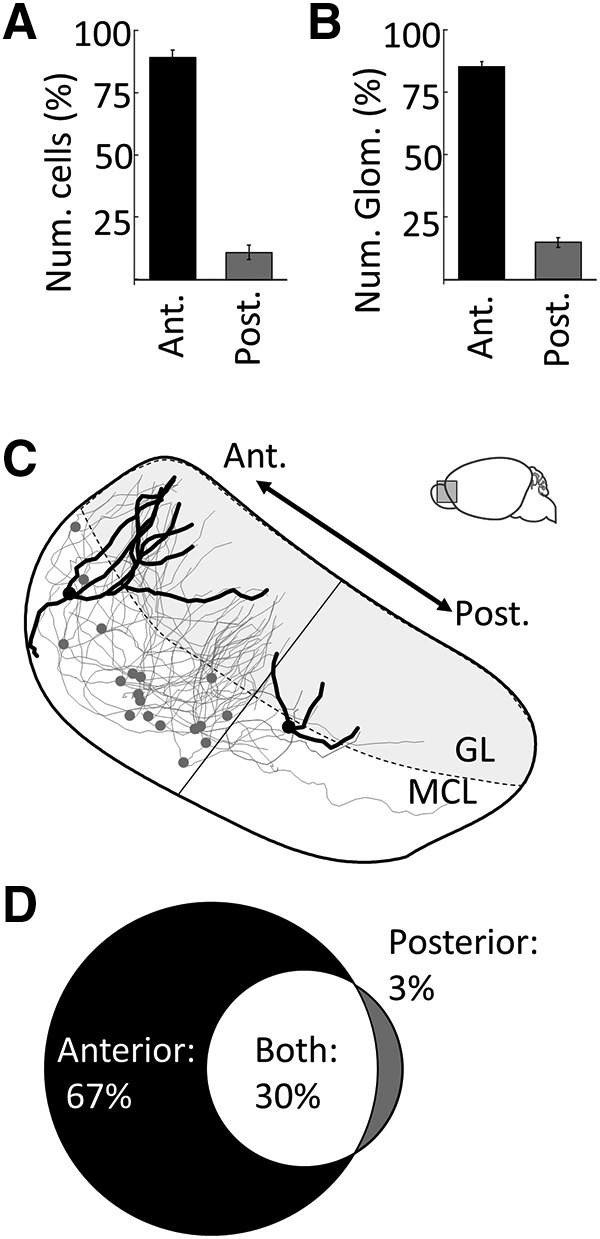
Quantitative analysis of AOB input to arom^+^ neurons in the MeA. ***A***, Percentage of →arom^+^ neuron cell bodies in the anterior versus posterior AOB. ***B***, Percentage of →arom^+^ glomeruli in the anterior versus posterior AOB. ***C***, Neuroanatomical reconstruction of the all →arom^+^ in the MeA of a single female mouse. Two neurons are highlighted: the first receives input only from anterior glomeruli, while the second, with a markedly smaller dendritic arbor, receives input from only posterior glomeruli (linea alba, thin straight line). ***D***, The fraction of →arom^+^ neurons that receive input from only the anterior AOB glomeruli (black), from only the posterior AOB glomeruli (gray), or from both the anterior and posterior AOB glomeruli (white). Error bars indicate SEM.

**Figure 5.**
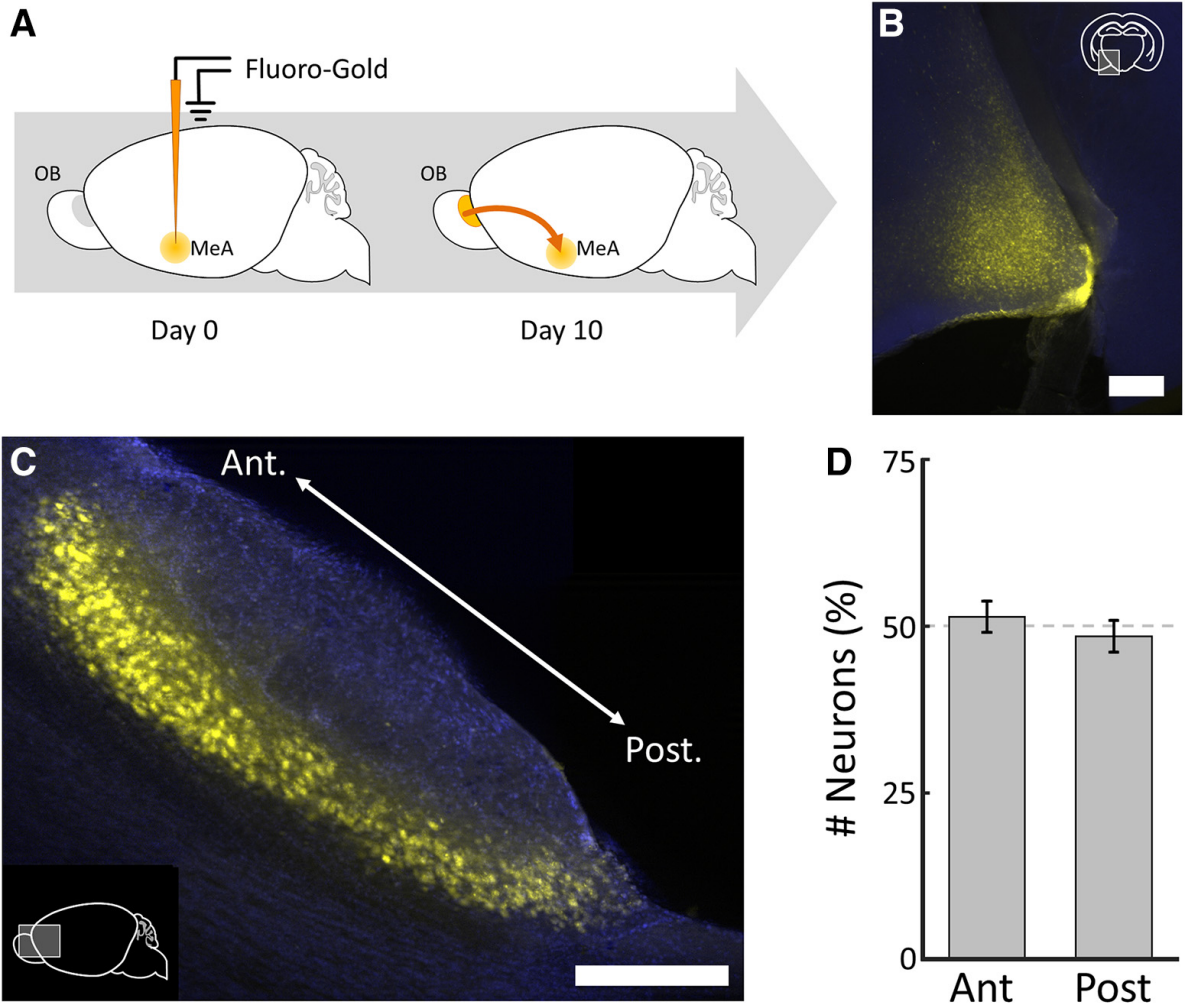
Classical retrograde tracing from the MeA to the AOB. ***A***, Fluoro-Gold was iontophoretically injected into the MeA, and 10 d were allowed for retrograde transport of Fluoro-Gold from the MeA to the AOB. ***B***, A Fluoro-Gold injection site demonstrates labeling in both the dorsal and ventral MeA. ***C***, Retrogradely labeled projection neurons in the AOB mitral cell layer showing dense labeling in both the anterior and posterior AOB. ***D***, Percentage of neurons located in the anterior versus posterior AOB. Scale bars, 250 μm.

Imaging the intact AOB using CLARITY and lightsheet microscopy (see Materials and Methods) allowed identification of nearly all →arom^+^ neurons and their corresponding glomeruli (Fig. 3*D*). The linea alba was used to distinguish the anterior AOB from the posterior AOB, and the distribution of →arom^+^ neurons was heavily skewed to the anterior AOB (Fig. 4*A*; anterior, 89 ± 2.9%; posterior, 11 ± 2.9%; unpaired *t* test: *p* < 0.00,001, *t* = 17.6, df = 20; Table 1, h). In contrast to the anterior AOB bias observed for rabies-infected arom^+^-projecting neurons, nearly all AOB mitral and tufted cells in both the anterior and posterior AOBs were labeled by the nonconditional retrograde tracer Fluoro-Gold iontophoretically injected in the MeA (Fig. 5; anterior, 51 ± 2.4%; posterior, 49 ± 2.4%; unpaired *t* test: *p* = 0.41, *t* = 0.88, df = 8; Table 1, i). Thus, both anterior and posterior AOB neurons project to the MeA, but arom^+^ MeA neurons receive input nearly exclusively from the anterior AOB.

The dendritic arbors of →arom^+^ neurons in the AOB were traced to the glomeruli they innervated. The →arom^+^ neurons predominantly innervated anterior AOB glomeruli that receive vomeronasal 1 receptor (V1R)-derived sensory information from the VNO. We imaged the dendritic arbors of →arom^+^ AOB neurons, including the innervated glomeruli, in a subset of animals (Fig. 4*B*,*C*). The dendrites of each →arom^+^ neuron in the AOB ramified in 2–10 glomeruli with an average of 4.2 glomeruli innervated per cell. The vast majority of glomerular inputs came from the anterior AOB (Fig. 4*B*; anterior, 85 ± 1.9%; anterior, 15 ± 1.9%; unpaired *t* test: *p* < 0.00,001, *t* = 21.4, df = 16; Table 1, j). Sixty-seven percent of →arom^+^ neurons had dendrites that innervated only anterior AOB glomeruli; 30% of the labeled AOB neurons had dendrites that innervated anterior and posterior AOB glomeruli; and 3% of the labeled AOB neurons had dendrites that innervated only posterior AOB glomeruli (Fig. 4*D*). Thus, the segregation between anterior and posterior AOBs is not complete; some integration occurs between anterior and posterior glomeruli even within the AOB. Of the AOB neurons receiving input from the posterior glomeruli, 91% also received input from at least one anterior glomerulus, and most received input from more anterior than posterior glomeruli.

**Figure 6.**
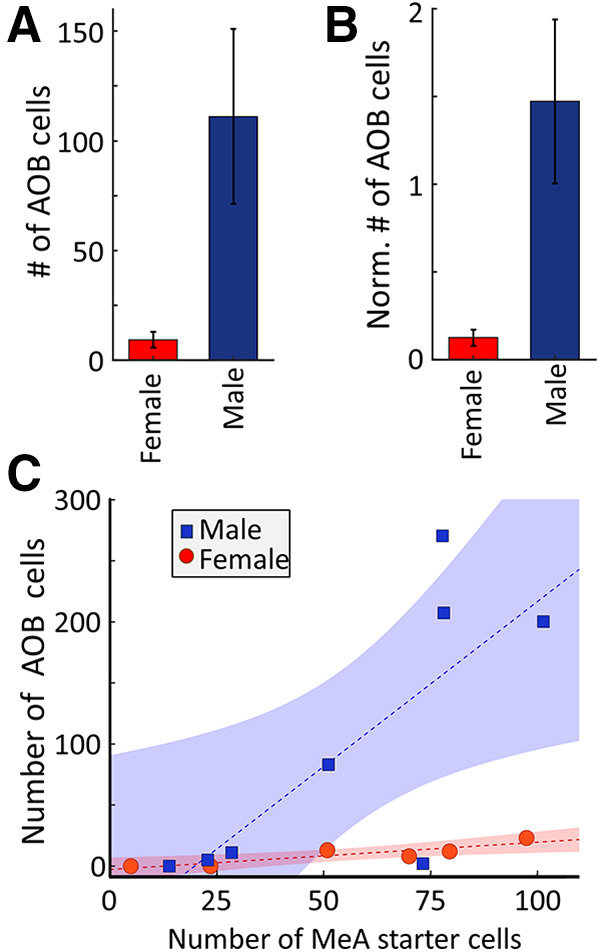
Sex differences in the AOB projection to arom^+^ MeA neurons. ***A***, The average number of arom^+^ projecting AOB neurons in female (red) or male (blue) mice. ***B***, Average number of arom^+^ projecting AOB neurons in female (red) or male (blue) mice after normalization by the number of starter neurons in the MeA. ***C***, Relationship between the numbers of arom^+^ starter neurons in the MeA and →arom^+^ neurons in the AOB of male (blue squares) and female (red circles) mice. Dashed lines indicate the best linear fit to the data, and the shaded regions indicate the 95% confidence interval on each fit. Error bars indicate SEM.

This analysis also revealed a stark sex difference. More →arom^+^ neurons were labeled by retrograde tracing in the AOB of male mice compared with female mice (Fig. 6). In both male and female mice, the number of infected AOB neurons was proportional to the number of arom^+^ “starter neurons” infected in the MeA (Fig. 6*C*; female regression: *R*^2^ = 0.80, *p* = 0.016; male regression: *R*^2^ = 0.63, *p* = 0.018). Because there are a larger number of aromatase neurons in the male MeA (Stanić et al., 2014), one possible explanation of the differential labeling observed in the AOB could be that more arom^+^ starter neurons were initially infected in males. While we found a small difference in the numbers of arom^+^ starter neurons in the male than in the female MeA, this difference was not statistically significant (male, 56.6 ± 11.2 MeA cells; female, 54.5 ± 14.3 MeA cells; unpaired *t* test: *p* = 0.93, *t* = 0.08, df = 12; Table 1, k).

To further rule out the possibility that the larger number of neurons labeled in the AOB of males was due to slight differences in the numbers of starter neurons, we normalized the number of labeled AOB projection neurons by dividing by the number of starter neurons in the MeA of each animal (Fig. 6*B*). The stronger labeling in the male AOB remained significant after accounting for differences in the number of starter neurons in each animal (male, 1.25 ± 0.43 AOB cells/MeA cell; female, 0.13 ± 0.05 AOB cells/MeA cell; unpaired *t* test: *p* = 0.04, *t* = 2.22, df = 12; Table 1, l). Comparing the pattern of connectivity between the AOB and MeA arom^+^ neurons in male and female mice revealed a similar anterior bias in both sexes, with the vast majority of labeled neurons located in the anterior AOB (anterior cells in males, 86.068 ± 4.0%; anterior cells in females, 93.9 ± 3.3%;, unpaired *t* test: *p* = 0.24, *t* = 1.2, df = 9, Table 1, m). A regression analysis comparing the rate of AOB infection for the number of starter neurons infected in males and females showed a larger slope for males than females, with the 95% confidence intervals for predicted AOB neurons separated for MeA infection rates >50 starter neurons in a given 100 μm slice. Therefore, we found that each arom^+^ neuron in the male MeA receives input from nearly an order of magnitude more AOB neurons on average—indicating a robust sexual difference in the connectivity between the anterior AOB and aromatase-expressing neurons in the MeA (Fig. 6*C*).

## Discussion

Here, we show that in both male and female mice, a set of neurons in the AOB projects directly to arom^+^ neurons in the MeA. Approximately 90% of AOB input to arom^+^ neurons is from the anterior AOB and 10% is from the posterior AOB, and essentially no direct input is from the main olfactory bulb. The topographical segregation of V1R-derived and vomeronasal 2 receptor (V2R)-derived information established in the VNO is maintained in the AOB, as V1R-expressing sensory neurons project to the anterior AOB and V2R-expressing sensory neurons project to the posterior AOB (Dulac and Torello, 2003). It is possible that the enhancement of anterior AOB neurons projecting to arom^+^ neurons in the MeA may also reflect the topographic distribution of arom^+^ neurons in the posterodorsal MeA. Because arom^+^ MeA neurons receive input almost exclusively from the anterior AOB (V1R), our study demonstrates that the separation of V1R-derived from V2R-derived sensory channels is maintained at least to the level of the MeA. Consistent with these findings, behaviors mediated by arom^+^ MeA neurons (Unger et al., 2015; Yao et al., 2017) are likely driven by sensory cues detected by V1R receptors. V1R receptors respond to lipophilic odorants, including sulfated steroids, that convey information about the physiological status of conspecific individuals (Nodari et al., 2008; Isogai et al., 2011; Hammen et al., 2014), indicating that arom^+^ neurons have direct access to sensory channels strongly linked to sexually differentiated behaviors (Nodari et al., 2008; Isogai et al., 2011).

While the sensory stimuli and neuroendocrine states that drive sex differences in social behaviors have been established for some time, the circuit mechanisms that produce sex differences in behavior are less well understood. Initially, we hypothesized that differences in the intrinsic properties of arom^+^ neurons may produce sex differences in circuit functions (Cooke et al., 1999). This line of reasoning was based on the critical role for aromatase in the development and maintenance of hormone-dependent sex differences in MeA neuroanatomy (Cooke et al., 1999; Morris et al., 2008; Wu et al., 2009; Bergan et al., 2014). While we were able to establish that arom^+^ neurons represent an electrophysiologically distinct category of MeA neuron (Keshavarzi et al., 2014), we observed no obvious sex differences in the intrinsic properties of either the arom^+^ or arom^−^ neurons. The lack of clear sex differences in arom^+^ neurons in this region is, at first pass, surprising given known sex differences in anatomy, sensory representation, and contributions to the behavior of MeA neurons (Cooke et al., 1999; Morris et al., 2008; Wu et al., 2009; Bergan et al., 2014; Hong et al., 2014; Unger et al., 2015; Yao et al., 2017).

Our anatomic results demonstrate that some arom^+^ neurons receive input from the anterior AOB, however, the percentage of MeA arom+ neurons that receive AOB input is not yet known. Our electrophysiological experiments could not distinguish arom+ MeA neurons based on whether they did or did not receive AOB input and may have missed a subtle difference in a relevant subpopulation. Moreover, our electrophysiological data cannot rule out all potential sex differences in electrophysiological function of MeA neurons. For example, it is possible that the different hormonal milieus present in adult males and females, including those linked to estrus state, may induce differences in electrophysiological function that are not captured by the *ex vivo* slice preparation we used (Cooke et al., 1999; Woolley, 1999).

**Figure 7.**
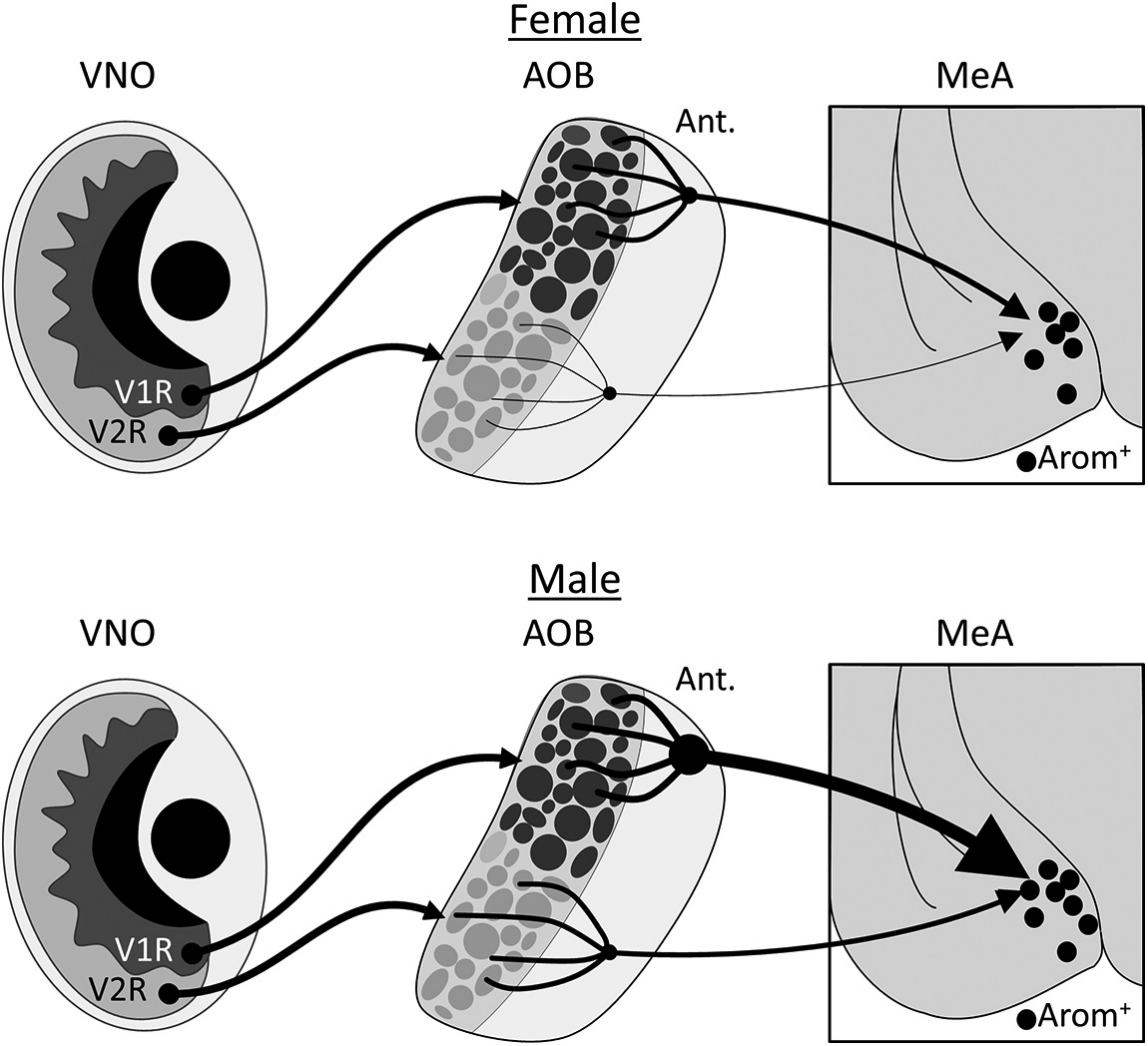
Diagram of AOB inputs to arom^+^ MeA neurons in male and female mice. V1R-expressing sensory neurons project from the VNO to the anterior AOB in both male and female mice, while V2R-expressing sensory neurons project to the posterior AOB. Mitral cells in the anterior AOB provide ∼90% of the input to arom^+^ neurons in the MeA, with the remaining 10% coming from the posterior AOB. This ratio is similar between male and female mice. The male MeA contains more aromatase neurons than the female MeA, each of which receives more input from the anterior AOB.

Sex differences in neural processing exist at many points along the trajectory from sensory transduction to motor output. Some moths express different receptors at the sensory epithelium of male and female animals (Schneiderman et al., 1986). Fruit flies display few sex differences at the sensory epithelium, but second-order projection neurons route sex-specific sensory information to different targets in the brains of male and female flies (Ryner et al., 1996; Ruta et al., 2010; Kohl et al., 2013). Similarly, we found that the second-order projection from AOB neurons to arom^+^ neurons in the MeA displays a large sex difference in mice (Fig. 7). The larger number of AOB neurons labeled in the male AOB cannot be explained by the larger number of arom^+^ neurons in the male MeA (Cooke et al., 1999) as only slightly more arom^+^ MeA starter neurons were infected in male mice. Moreover, the sex difference in AOB neurons projecting to arom^+^ MeA neurons persists even after normalizing by the size of the initial MeA infection. Therefore, the projection of →arom^+^ neurons to the MeA of mice is configured differently in male and female mice.

The functional consequences of the anatomic sex difference described here are difficult to predict. It is possible that AOB input to arom^+^ MeA neurons of male animals is simply stronger, with greater numbers of synaptic contacts. Another possibility is that there is less branching of AOB neurons in males and, accordingly, males require integration from a larger number of AOB neurons to innervate the same percentage of MeA arom^+^ neurons. Indeed, each individual AOB synapse with an arom^+^ MeA neuron may be weaker in male versus female mice. Moreover, while both male mice and female mice receive input from the anterior AOB, it is possible that the subset of V1R receptors providing sensory input is different in male versus female mice. Despite these outstanding questions, our results unambiguously show that the projection from AOB neurons to arom^+^ MeA neurons is configured differently in male versus female mice.

## Synthesis

Reviewing Editor: Margaret McCarthy, Univ of Maryland School of Medicine Decisions are customarily a result of the Reviewing Editor and the peer reviewers coming together and discussing their recommendations until a consensus is reached. When revisions are invited, a fact-based synthesis statement explaining their decision and outlining what is needed to prepare a revision will be listed below. The following reviewer(s) agreed to reveal their identity: Jacques Balthazart, Lance Kriegsfeld.

By a combination of electrophysiological and tract tracing studies, this paper demonstrates that aromatase-positive neurons in the male medial amygdala receive denser projections from the anterior AOB as compared to females. No major differences in electrophysiological properties or aromatase neurons were detected between males and females although aromatase-positive neurons displayed some electrophysiological characteristics that were different from the aromatase-negative neurons.

The medial amygdala is a key component of the social behavior network and is sexually dimorphic in its neurochemistry and structure. Because aromatase is expressed in a subset of neurons of the MeA, and sex steroid hormones regulate sexual differentiation of this neural locus and its adult function, the authors speculated that differences in olfactory input and electrophysiological properties of aromatase-expressing MeA neurons might account for sex differences in social behavior mediated by this sub nucleus. To explore this possibility, the authors crossed the Cyp19a1-Cre transgenic line to rosa26--lsl-tDTomato reporter mice to express Cre/tdTomato specifically in aromatase neurons. Using this strategy, the authors examined electrophysiological properties of aromatase-positive and -negative neurons in the MeA of males and females. Although sex differences in electrophysiological properties (e.g., input resistance, threshold to initiate action potentials, # of elicited action potentials) of aromatase-positive and -negative MeA neurons were not observed, a greater number of AOB inputs to these cells was identified in males compared to females. Of note, the vast majority of these inputs (in both sexes) arose from the anterior AOB, with essentially no input from the main olfactory bulb. This finding suggests that input to MeA aromatase-expressing cells arises from V1R-expressing sensory neurons given the topographic organization of the AOB. Together, these findings point to sex differences in the degree of AOB input to MeA aromatase-expressing neurons and identify this circuit as a target for further investigation in the study of sexually differentiated social behavior.

Statistics: Analyses are potentially correct but they are not reported with sufficient detail (see comments to authors)

Software comments: It is unclear whether validation was performed. Minimal details are provided for ‘custom’ scripts used in Matlab for image analysis.

Concerns about animal subjects: Not a concern, but the statement: “all animal procedures were performed in accordance with the [Author University] animal care committee regulations.” The statement should indicate that the procedures were APPROVED by the IACUC.

Multimedia comments: The movies were included help to visualize how the analyses were conducted and the extent of projections. we would support inclusion.

Detailed comments:

1) Line 8-9: This sentence of the abstract is misleading (We found that sensory input to aromatase neurons is derived nearly exclusively from the anterior AOB). Within the AOB the inputs come mainly from the anterior part but this study did not investigate other brain regions that could provide other sensory inputs. Please rephrase too stay closer to the data. The statement on lines 259-260 is more accurate in this respect.

2) Lines 175 and following: it is technically inappropriate to base a statistical analysis on the number of neurons. The true number of independent replications is the number of subjects, not the number of neurons since there is potentially a lack of independence between different neurons studied in the same subject. This remark is of little importance in the present case since you are reporting a lack of difference. Had you reported a difference, I would contend that the difference is not established in a valid manner. To reassure the reader it would be important to add in parentheses in each case (here and later in the text) the number of subjects that were used for each analysis and the degrees of freedom associated with the t tests.

3) Lines 185-194: The documentation of the numbers of spikes in the two sexes and two types of neurons is very (too) schematic even if the figure 2E is esthetically quite pleasing. Red and blue represent females and males respectively. The legend then mentions a shaded area but I only see an orange area and I am unable to relate it to the male of the female curve. Is it an area common to both? The figure would be more informative if the SEM was shown for each curve only above or below the curve that is concerned depending on its position. Also, the analysis was done by ANOVA; I assume this must be a two way ANOVA with the sex of subjects as independent factor and the injected current as repeated factor. The full results of this ANOVA then must include 2 F for the main factors and one F for the interactions. A complete description of the results (F, df, P) would be needed for each factor and interaction. It is not sufficient to say p=0.01 ANOVA. Finally how was the Cohen D calculated? This cannot be understood with the available information.

4) Line 210, and 222: Is this a test for repeated samples? The value of t and the degrees of freedom should be indicated

5) Lines 233-234, 239, 244-45, 248-249: please add the degrees of freedom and value of t.

6) Line 297: the projection is not dimorphic sensu stricto, it is sexually differentiated. Dimorphic= different forms. This language error is quite common but should probably be corrected. Editors Note: I agree with this statement.

7) On page 10, please indicate how normalization of data was accomplished. The hypothesis might also be considered that, in females, a smaller number of AOB cells innervate a similar percentage of MeA arom+ cells as seen in males (e.g., in males, there is less branching and more AOB cells required to innervate the same percentage of MeA arom+ cells).

8) Was the estrous cycle of females monitored and brains examined relative to a specific point in the ovulatory cycle? The lack of sex differences observed in electrophysiological properties may be affected by variability in females.

9) Was time of day considered when brains were examined as daily variability in function may cloud interpretation of electrophysiological findings.

10) Both reviewers felt this point was important - It is unclear how the approach of using AAV-FLEX-TVA in the absence of AAV-FLEX-RG was used to confirm the specificity of starter neurons. Aromatase positive cells express tdTomato and it is assumed the AAV-FLEX-TVA expresses mCherry. The results do not present any information on co-localization of TVA receptor/aromatase. Was mCherry stained immunohistochemically with a ‘green’ fluorophore? If so, images showing the double-label should be included in Figure 3.

11) The reviewers and Rev Editor agreed that the following experiment is not necessary since eNeuro does not require mechanism but it is included as an FYI - Because the results suggest that males might have greater AOB input to MeA arom+ cells, it might be useful to have an experiment in which a ChR2-expressing AAV is injected into the anterior AOB and aromatase-expressing cells are examined with and without light stimulation. This would allow a functional determination of the significance of the greater input to male MeA arom+ cells.

12) Reviewers also emphasized this point - It would be useful to show the extent of FG spread relative to MeA arom+ cells in Figure 5B to see whether or not projections from the posterior AOB arise due to significant spread beyond the region containing arom+ cells.

13) Discussion: I understand that the main focus of this paper is on sex differences but it might also be interesting to say a few words in the discussion about the difference between aromatase-positive and aromatase-negative neurons that produced different numbers of spikes in response to the same stimulation. Could this have any physiological meaning? It was surprising not to see neuronal physiology conducted in the presence/absence of sex steroids given their prominent role in MeA-mediated social behavior. Is it possible you are missing sex differences in neuronal physiology by not dissociating those arom+ cells that do/do not receive AOB input and differences that might emerge when sex steroids are present? They already mention that different results might be obtained with sex steroids in the Discussion. So, perhaps you can also consider the possibilities (in the Discussion) that investigating arom+ cells that do/do not receive AOB input, or investigating arom+ cells under conditions of AOB pathway stimulation, may reveal sex differences.

Minor

Line 77 : the word “ CO2” after the parenthesis should probably be deleted

Line 79 and 82: replace “/H20” with “in H20"

Line 132 delete “of” after “into the “MeA"

Line 208: Would it be possible to mark the linea alba in figure 3D? Is it the line separating the white and gray areas in figure 4C? (if so please mention in the legend for readers not familiar with the AOB anatomy)

On page 3, Figure 1C should be Figure 2C

Please indicate the age range of mice.

One reviewer suggested eliminating Figure 1 as this is described well enough in the text. However since this an on-line journal there are no limits on figures so it is at your discretion

## References

[B1] Bergan JF, Ben-shaul Y, Dulac C (2014) Sex-specific processing of social cues in the medial amygdala. Elife 3:e02743 10.7554/eLife.02743 24894465 PMC4038839

[B2] Chung K, Wallace J, Kim S-Y, Kalyanasundaram S, Andalman AS, Davidson TJ, Mirzabekov JJ, Zalocusky KA, Mattis J, Denisin AK, Pak S, Bernstein H, Ramakrishnan C, Grosenick L, Gradinaru V, Deisseroth K (2013) Structural and molecular interrogation of intact biological systems. Nature 497:332–337. 10.1038/nature12107 23575631 PMC4092167

[B3] Cooke BM, Tabibnia G, Breedlove SM (1999) A brain sexual dimorphism controlled by adult circulating androgens. Proc Natl Acad Sci U S A 96:7538–7540. 10.1073/pnas.96.13.7538 10377450 PMC22121

[B4] Dulac C, Torello AT (2003) Molecular detection of pheromone signals in mammals: from genes to behaviour. Nat Rev Neurosci 4:551–562. 10.1038/nrn1140 12838330

[B5] Ferguson JN, Aldag JM, Insel TR, Young LJ (2001) Oxytocin in the medial amygdala is essential for social recognition in the mouse. J Neurosci 21:8278–8285. 11588199 10.1523/JNEUROSCI.21-20-08278.2001 PMC6763861

[B6] Goodson JL (2005) The vertebrate social behavior network: evolutionary themes and variations. Horm Behav 48:11–22. 10.1016/j.yhbeh.2005.02.003 15885690 PMC2570781

[B7] Hammen GF, Turaga D, Holy TE, Meeks JP (2014) Functional organization of glomerular maps in the mouse accessory olfactory bulb. Nat Neurosci 17:953–961. 10.1038/nn.3738 24880215 PMC4327767

[B8] Hong W, Kim DW, Anderson DJ (2014) Antagonistic control of social vs repetitive self-grooming behaviors by separable amygdala neuronal subsets. Cell 158:1348–1361. 10.1016/j.cell.2014.07.049 25215491 PMC4167378

[B9] Isogai Y, Si S, Pont-Lezica L, Tan T, Kapoor V, Murthy VN, Dulac C (2011) Molecular organization of vomeronasal chemoreception. Nature 478:241–245. 10.1038/nature10437 21937988 PMC3192931

[B10] Isogai Y, Richardson D, Dulac C, Bergan JF (2017) Optimized protocol for imaging cleared neural issues using light microscopy In: Synapse development, methods and protocols. (Poulopoulos A, ed), pp 137–153. New York: Springer. 10.1007/978-1-4939-6688-2_11 27943189

[B11] Keshavarzi S, Sullivan RKP, Ianno DJ, Sah P (2014) Functional properties and projections of neurons in the medial amygdala. J Neurosci 34:8699–8715. 10.1523/JNEUROSCI.1176-14.2014 24966371 PMC6608208

[B12] Kohl J, Ostrovsky AD, Frechter S, Jefferis GS (2013) A bidirectional circuit switch reroutes pheromone signals in male and female brains. Cell 155:1610–1623. 10.1016/j.cell.2013.11.025 24360281 PMC3898676

[B13] Lehman MN, Winans SS, Powers JB (1980) Medial nucleus of the amygdala mediates chemosensory control of male hamster sexual behavior. Science 210:557–560. 10.1126/science.7423209 7423209

[B14] Madisen L, Zwingman TA, Sunkin SM, Oh SW, Zariwala HA, Gu H, Ng LL, Palmiter RD, Hawrylycz MJ, Jones AR, Lein ES, Zeng H (2010) A robust and high-throughput Cre reporting and characterization system for the whole mouse brain. Nat Neurosci 13:133–140. 10.1038/nn.2467 20023653 PMC2840225

[B15] Mandiyan VS, Coats JK, Shah NM (2005) Deficits in sexual and aggressive behaviors in Cnga2 mutant mice. Nat Neurosci 8:1660–1662. 10.1038/nn1589 16261133

[B16] Menegas W, Bergan JF, Ogawa SK, Isogai Y, Umadevi Venkataraju K, Osten P, Uchida N, Watabe-Uchida M (2015) Dopamine neurons projecting to the posterior striatum form an anatomically distinct subclass. Elife 4:e10032. 10.7554/eLife.10032 26322384 PMC4598831

[B17] Morris JA, Jordan C, Breedlove SM (2008) Sexual dimorphism in neuronal number of the posterodorsal medial amygdala is independent of circulating androgens and regional volume in adult rats. J Comp Neurol 506:851–859. 10.1002/cne.21536 18076082

[B18] Newman SW (1999) The medial extended amygdala in male reproductive behavior: a node in the mammalian social behavior network. Ann N Y Acad Sci 877:242–257. 10.1111/j.1749-6632.1999.tb09271.x 10415653

[B19] Nodari F, Hsu FF, Fu X, Holekamp TF, Kao LF, Turk J, Holy TE (2008) Sulfated steroids as natural ligands of mouse pheromone-sensing neurons. J Neurosci 28:6407–6418. 10.1523/JNEUROSCI.1425-08.2008 18562612 PMC2726112

[B20] Petrovich GD, Canteras NS, Swanson LW (2001) Combinatorial amygdalar inputs to hippocampal domains and hypothalamic behavior systems. Brain Res Brain Res Rev 38:247–289. 10.1016/s0165-0173(01)00080-7 11750934

[B21] Ryner LC, Goodwin SF, Castrillon DH, Anand A, Villella A, Baker BS, Hall JC, Taylor BJ, Wasserman SA (1996) Control of male sexual behavior and sexual orientation in Drosophila by the fruitless gene. Cell 87:1079–1089. 10.1016/s0092-8674(00)81802-4 8978612

[B22] Ruta V, Datta SR, Vasconcelos ML, Freeland J, Looger LL, Axel R (2010) A dimorphic pheromone circuit in Drosophila from sensory input to descending output. Nature 468:686–690. 10.1038/nature09554 21124455

[B23] Schneiderman AM, Hildebrand JG, Brennan MM, Tumlinson JH (1986) Trans-sexually grafted antennae alter pheromone-directed behaviour in a moth. Nature 323:801–803. 10.1038/323801a0 3774007

[B24] Stanić D, Dubois S, Chua HK, Tonge B, Rinehart N, Horne MK, Boon WC (2014) Characterization of aromatase expression in the adult male and female mouse brain: I. Coexistence with oestrogen receptors α and β, and androgen receptors. PLoS One 9:e90451. 10.1371/journal.pone.0090451 24646567 PMC3960106

[B25] Stowers L, Holy TE, Meister M, Dulac C, Koentges G (2002) Loss of sex discrimination and male-male aggression in mice deficient for TRP2. Science 295:1493–1500. 10.1126/science.1069259 11823606

[B26] Tinbergen N (1951) The study of instinct. New York: Oxford UP.

[B27] Unger EK, Burke KJ Jr, Yang CF, Bender KJ, Fuller PM, Shah NM (2015) Medial amygdalar aromatase neurons regulate aggression in both sexes. Cell Rep 10:453–462. 10.1016/j.celrep.2014.12.040 25620703 PMC4349580

[B28] Watabe-Uchida M, Zhu L, Ogawa SK, Vamanrao A, Uchida N (2012) Whole-brain mapping of direct inputs to midbrain dopamine neurons. Neuron 74:858–873. 10.1016/j.neuron.2012.03.017 22681690

[B29] Wickersham IR, Lyon DC, Barnard RJ, Mori T, Finke S, Conzelmann KK, Young JA, Callaway EM (2007) Monosynaptic restriction of transsynaptic tracing from single, genetically targeted neurons. Neuron 53:639–647. 10.1016/j.neuron.2007.01.033 17329205 PMC2629495

[B30] Woolley CS (1999) Electrophysiological and cellular effects of estrogen on neuronal function. Crit Rev Neurobiol 13:1–20. 10.1615/critrevneurobiol.v13.i1.10 10223521

[B31] Wu MV, Manoli DS, Fraser EJ, Coats JK, Tollkuhn J, Honda S, Harada N, Shah NM (2009) Estrogen masculinizes neural pathways and sex-specific behaviors. Cell 139:61–72. 10.1016/j.cell.2009.07.036 19804754 PMC2851224

[B32] Yao S, Bergan J, Lanjuin A, Dulac C (2017) Oxytocin signaling in the medial amygdala is required for sex discrimination of social cues. Elife 6:e31373 10.7554/eLife.31373 29231812 PMC5768418

